# Multi-Task Learning Based Network Embedding

**DOI:** 10.3389/fnins.2019.01387

**Published:** 2020-01-14

**Authors:** Shanfeng Wang, Qixiang Wang, Maoguo Gong

**Affiliations:** ^1^School of Cyber Engineering, Xidian University, Xi'an, China; ^2^Key Laboratory of Intelligent Perception and Image Understanding of Ministry of Education, School of Electronic Engineering, Xidian University, Xi'an, China

**Keywords:** network representation learning, multi-task learning, network embedding, high-order proximity, low-order proximity

## Abstract

The goal of network representation learning, also called network embedding, is to encode the network structure information into a continuous low-dimensionality embedding space where geometric relationships among the vectors can reflect the relationships of nodes in the original network. The existing network representation learning methods are always single-task learning, in which case these methods focus on preserving the proximity of nodes from one aspect. However, the proximity of nodes is dependent on both the local and global structure, resulting in a limitation on the node embeddings learned by these methods. In order to solve this problem, in this paper, we propose a novel method, Multi-Task Learning-Based Network Embedding, termed MLNE. There are two tasks in this method so as to preserve the proximity of nodes. The aim of the first task is to preserve the high-order proximity between pairwise nodes in the whole network. The second task is to preserve the low-order proximity in the one-hop area of each node. By jointly learning these tasks in the supervised deep learning model, our method can obtain node embeddings that can sufficiently reflect the roles that nodes play in networks. In order to demonstrate the efficacy of our MLNE method over existing state-of-the-art methods, we conduct experiments on multi-label classification, link prediction, and visualization in five real-world networks. The experimental results show that our method performs competitively.

## 1. Introduction

A network is an important way of representing the relationships between objects, for example, in social networks, state grids, and citation networks (Gong et al., 2017). With the increasing complexity of a network, it is more valuable to explore it as a carrier of information. There are some meaningful applications in network analysis, such as node classification (Tsoumakas and Katakis, 2007), link prediction (Lü and Zhou, 2011), community detection (Fortunato, 2010), and recommender systems (Lü et al., 2012). Traditional network representation methods, such as an adjacency matrix, pose several challenges (Peng et al., 2019). First, network analysis methods based on traditional forms of representation usually have high computational complexity. Second, traditional network representation methods make it difficult to design parallel and distributed algorithms. These two challenges make these methods hard to use for large-scale network analysis. Moreover, there is a limitation when machine learning is applied in network analysis due to high dimensionality and sparsity. Thus, determining how to properly construct a meaningful representation of the structure information extracted from networks is promising research.

Network representation learning (NRL), also called network embedding (Hamilton et al., 2017; Goyal and Ferrara, 2018), has been proposed for encoding network information into a continuous low-dimensionality feature space. From the perspective of network topology, those nodes that have similar structures should have similar representation vectors. For example, those nodes within the same community in a network have similar proximity structures, and thus they should be closer in embedding space. Due to the learned representations, the relationships between nodes and the roles that nodes play in networks can be efficiently analyzed. Many network analysis tasks can be dealt with based on the distances in the embedding space, so that the computational complexity is low and parallel algorithms can be adopted for network analysis problems. Moreover, many machine-learning algorithms have been used for network analysis, benefiting from network embedding. Not only that, but those representations can be applied in other application tasks (Herman et al., 2000; Hu et al., 2016; Wang et al., 2017; Wei et al., 2017; Shi et al., 2018).

Recently, an increasing number of methods have been proposed for network representation learning (Chen et al., 2018; Peng et al., 2019; Zhang et al., in press). These methods can mainly be classified into three categories (Peng et al., 2019). The first is matrix factorization-based methods (Qiu et al., 2018; Liu et al., 2019b), which are directly inspired by the dimension-reduction technique. One of the best-known methods is Laplacian Eigenmaps (Belkin and Niyogi, 2002), which generate a network representation through factorizing the Laplacian of the network adjacency matrix. GraRep (Cao et al., 2015) builds a *k*-step relationship information matrix so as to sufficiently capture the pairwise node proximity. According to the matrix, it adopts SVD to generate different representations and finally concatenates all of them to form a global representation. Qiu et al. exploited sparse matrix factorization for large-scale network embedding (Qiu et al., 2019). The second category is random walk-based methods. DeepWalk (Perozzi et al., 2014) was the first method to introduce random walk into network representation learning. It uses a sampling method called unbiased random walk to generate discrete sequences of nodes, in which case sequences and nodes are abstracted as sentences and words. It also introduces the skip-gram (Mikolov et al., 2013), the best-known model in natural language processing (NLP), to learn representations for nodes from those sequences. Node2vec (Argerich et al., 2016) was proposed to develop a novel sampling method named biased random walk, which is based on breadth-first search (BFS) and depth-first search (DFS), resulting in more flexibility in the exploration of networks. The third category is deep learning-based methods. Wang et al. proposed a structural deep network embedding method named SDNE (Wang et al., 2016). Cao et al. proposed a deep neural network for learning graph representations (DNGR) (Cao et al., 2016). Both SDNE and DNGR follow the encoder-decoder framework, where the encoder maps a high-dimensionality feature vector into a lower-dimensionality representation and the decoder reconstructs the original feature vector from that. They build a proximity matrix in which an element represents the pairwise node proximity and apply an autoencoder model to learn representations from that matrix. SDNE directly adopts a network adjacency matrix as the proximity matrix and combines the autoencoder loss function with the Laplacian Eigenmaps loss function. DNGR introduces the pointwise mutual information (PMI) matrix as the proximity matrix, which is mostly used to evaluate the similarity among words in NLP. Network embedding methods are not limited to the above three categories (Tang et al., 2015; Donnat et al., 2018).

Existing network embedding algorithms have achieved promising performance, but these methods all focus on single-task learning, resulting in a lack of diversity in representations. A good representation of a node should depend on its position and structure in the local community and global network. For example, a node may be the centroid of the local community and also play a role as a bridge between communities in the global network (Musiał and Juszczyszyn, 2009). To learn network representation from both the local and global network structure information, we resort to multi-task learning (MTL) for help in exploring and exploiting global and local network representation learning.

In this paper, we propose a multi-task learning-based network embedding called MLNE. In MLNE, there are three components: a shared encoder, decoder, and classifier. We adopt *positive pointwise mutual information* (PPMI), which is a commonly used method to measure the similarity between discrete objects, to build the global proximity matrix. In order to build the matrix, we introduce random surfing to gather graph information. The shared encoder and decoder form a standard autoencoder to learn the latent representation from the global proximity matrix in an unsupervised manner. The shared encoder encodes the global feature information into a low-dimensionality node embedding, and the decoder decodes that information from the learned embeddings. Another task is to preserve the local features. The key idea behind this task is that the learned embedding from the shared encoder contains graph information such as the structure of local graph neighborhoods, so that the one-hop area of nodes can be reconstructed from that learned embedding. Due to the network sparsity, the direct neighborhoods should make more contributions to nodes, and thus it is worth designing a specific task to optimize embedding with respect to first-order proximity. The task is carried out by the shared encoder and the specific classifier, which predicts whether there is an edge between pairwise nodes. As a result, the learned embeddings can preserve both the local and global structural information. In addition, we design a regularizer to make those nodes that are direct neighborhoods for each other much closer in Euclidean space and vice versa, resulting in good clustering. Empirically, we conduct experiments on five real-world network datasets and three tasks: node classification, link prediction, and visualization. The experimental results show that our model has competitive performance against baselines.

The rest of this paper is organized as follows. In section II, preliminaries are given. Section III introduces the proposed algorithm in detail. In section IV, we briefly compare our algorithm with other related network embedding methods and analyze the experimental results. In the last section, this paper is concluded.

## 2. Preliminaries

In this section, we discuss the preliminaries of network representation learning in detail. First, we briefly introduce the notation and formulate the problem. Second, detailed descriptions of positive pointwise mutual information and random surfing are presented. An introduction to multi-task learning is then given.

### 2.1. Notations and Definitions

A network can be formally modeled as a graph *G* = (*V, E*), where *V* is the set of nodes and *E* is the set of edges. *v* ∈ *V* represents a node in the graph, and (*v*_*i*_, *v*_*j*_) ∈ *E* represents an edge between *v*_*i*_ and *v*. The adjacency matrix is defined as *A* ∈ ℝ^|*V*| × |*V*|^. Network representation learning aims to build an embedding matrix *Z* ∈ ℝ^|*V*| × *d*^, where *d* ≪|*V*| and each row *z* ∈ ℝ^*d*^ represents a vector representation of a node.

### 2.2. PPMI and Random Surfing

Pointwise mutual information (PMI) is a measure to quantify the correlation between two discrete objects. PMI has commonly been applied in the field of NLP such as in the measurement of the similarity between words. PMI can be defined as follows:

(1) $$\begin{array}{r} PMI\left(w,c\right)=\text{log}\left(\frac{\#\left(w,c\right)\cdot D}{\#\left(w\right)\cdot \#\left(c\right)}\right) \end{array}$$

where #(·) means the number of occurrences of an object and $D=\underset{w}{\sum }\underset{c}{\sum }\#\left(w,c\right)$.

It is found that when the statistics of co-occurrence count between two objects #(*w, c*) is 0, the measure will result in log(0) = −∞. An alternative measure called positive pointwise mutual information (PPMI) is proposed to address this problem. PPMI can be defined as follows:

(2) $$\begin{array}{r} PPMI\left(w,c\right)=\text{max}\left(0,PMI\left(w,c\right)\right) \end{array}$$

Cao et al. firstly introduced PPMI into NRL to generate node representation (Cao et al., 2016). In order to build PPMI matrix, they designed a random surfing model to extract structure information of network and directly generate the probabilistic co-occurrence matrix without sampling process. The key idea behind the model is that the visited probability from source node to target node can be iteratively calculated by a transition matrix.

Let the *P*_*k*_ be the *k*-th step visited probability matrix in which each element *P*_*k*_(*i, j*) represents the probability from source node *v*_*i*_ to node *v*_*j*_ after *k* times transitions. The *P*_0_ initially is set as *A*. The *P*_*k*_ can be defined as follows:

(3) $$\begin{array}{r} P_{k}=\gamma \cdot P_{k-1}\cdot T+\left(1-\gamma \right)\cdot P_{0} \end{array}$$

where *T* is the transition matrix, γ is the probability that the model will continue simulation, and 1 − γ is the restart probability. The element in *T* is the probability that node *v*_*i*_ will reach node *v*_*j*_. If *A*_*i,j*_ = 1, *T*(*i, j*) = 1/*deg*(*i*), otherwise *T*(*i, j*) = 0.

According to (3), a set of visited probability matrices can be defined as *P* = {*P*_0_, *P*_1_, …, *P*_*K*_}. The probabilistic matrix can be constructed as follows:

(4) $$\begin{array}{r} r=\overset{K}{\underset{i=k}{\sum }}P_{i}. \end{array}$$

where *K* is the number of samplings.

### 2.3. Multi-Task Learning

Traditional machine learning methods aim to optimize for a specific metric. To realize the goal of a task, a model is trained by fine-tuning parameters. By training the model, we can get a satisfying result, but some information that helps to improve the performance will be ignored. This information can be mined from related tasks. To utilize the information effectively, a new approach, named multi-task learning (MTL) (Ruder, 2017; Thung and Wee, 2018), is proposed. In MTL, multiple related tasks are learnt jointly, and useful information is shared among related tasks. In MTL, each task can benefit from other tasks, and then we can get a better result by training several tasks. Multi-task learning has been widely used in several fields, such as natural language processing (Liu et al., 2019a), image processing (Du et al., 2018), computer vision (Zhang et al., 2018), and recommendation (Wang et al., 2018).

**Figure 1:**
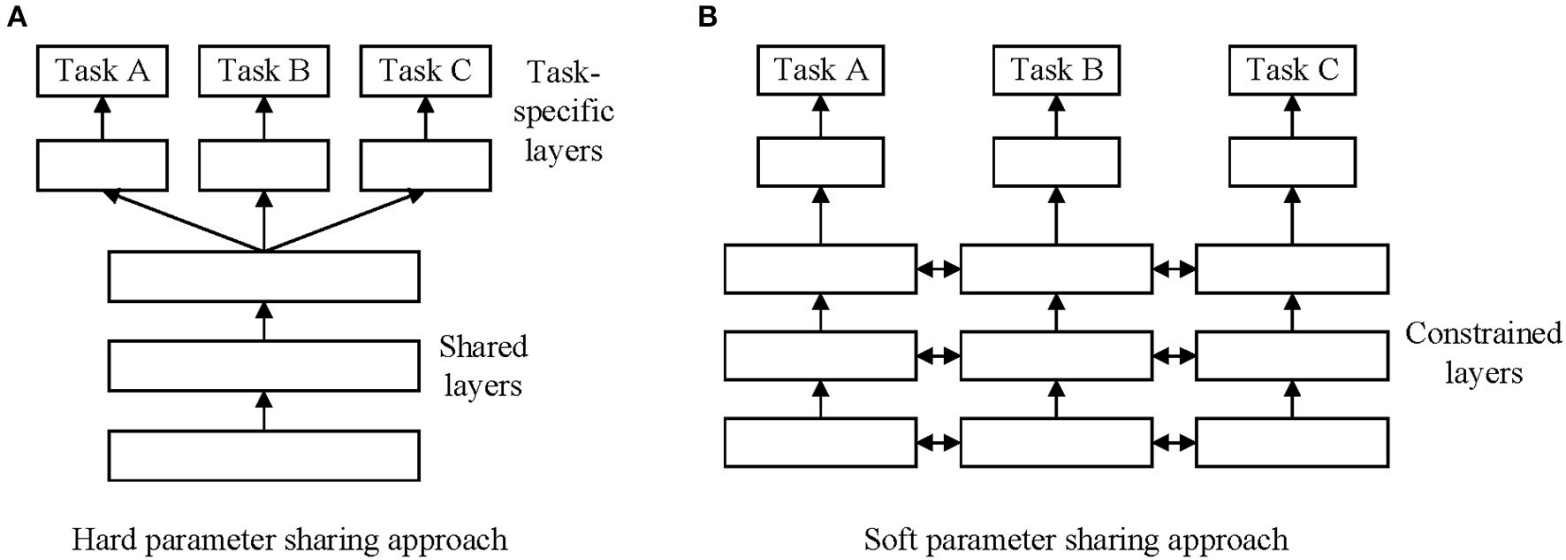
Two approaches for multi-task learning in deep neural networks. **(A)** Hard parameter sharing approach. **(B)** Soft parameter sharing approach.

There are two commonly used approaches to carrying out MTL in deep learning. The first is hard parameter sharing of hidden layers. In this approach, different tasks share the hidden layers, and the output layers are different. The second is soft parameter sharing of hidden layers. In this approach, different tasks have similar parameters, and the output layers are also different. Figure 1 shows these two approaches to MTL in deep learning.

## 3. The Framework

In this section, we first give the detailed description of the framework of our proposed approach, MLNE. Next, our multi-task learning model based on deep learning is described in detail.

### 3.1. An Overview of the Framework

**Figure 2:**
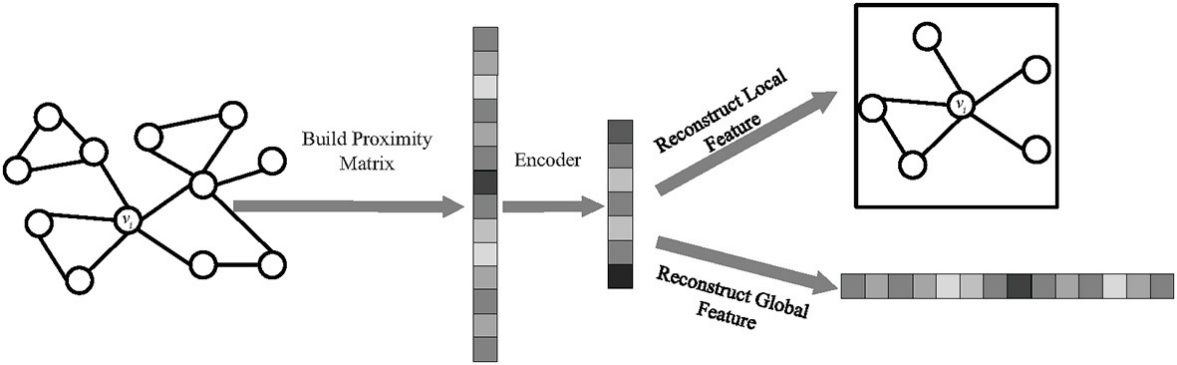
The framework of MLNE.

In this work, we leverage multi-task learning to learn robust and meaningful node representations. Figure 2 shows the framework of our proposed model, MLNE, in which there are two phases: building the proximity matrix and embedding nodes. In the first phase, we extract information on the local and global structures to build a proximity matrix where each element represents the similarity between nodes. In the second phase, our model jointly optimizes two tasks so as to learn node representations in which there are two tasks, preserving the global and local network structures. The framework of the proposed algorithm is given as Algorithm 1.

**Algorithm 1:** Framework of the proposed MLNE


**Input:** Input Graph: *G* = (*V, E*); Adjacency matrix: *A*; Number of samplings: *K*; Probability of resampling: γ; Weighted parameters of the loss function: α, β, η; Number of dimensions of representation vectors: *R*.
**Output:** Representation vectors of nodes: Φ.
1: Initialize matrix of node representations Φ ∈ ℝ^|*V*| × |*d*|^.
2: Construct the global proximity matrix *S*_*global*_;
3: Local proximity *S*_*local*_ ← *A*;
4: Initialize the parameters of the network: θ;
5: Input *S*_*global*_ into the neural network and train the network model by optimizing the objective function (Equation 10) by stochastic gradient descent.

### 3.2. Multi-Task Learning Model

**Figure 3:**
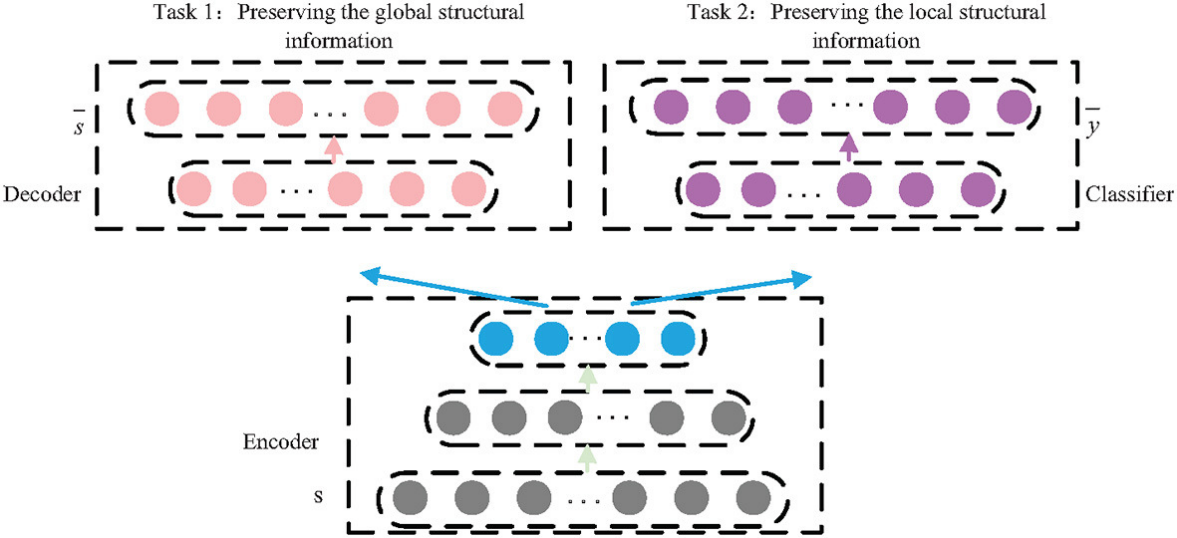
The architecture of MLNE.

Deep learning is introduced into multi-task learning model so as to learn complex structural information. In our proposed model, there are multiple layers with non-linear activation functions, such as *sigmoid* and *relu*, so as to build non-linear projections. At a high level, our model consists of three components as shown in Figure 3: a shared encoder network, a decoder network, and a classifier network.

The first task includes the shared encoder and the specific decoder and can be seen as a standard autoencoder model. The encoder maps the high-dimensionality structural information into a lower-dimensionality embedding space, *s*_*i*_ → *z*_*i*_, and the decoder reconstructs the structural information from the learned embeddings, $z_{i}\to {\bar{s}}_{i}$. In order to preserve the global structural information, the PPMI matrix is adopted as the global proximity matrix *S*, and a random surfing model is used to build the PPMI. The loss function can be defined as follows:

(5) $$\begin{array}{r} L_{global}=\overset{n}{\underset{i=1}{\sum }}||s_{i}-\bar{s_{i}}|{|}_{2}=||S-\bar{S}|{|}_{2}. \end{array}$$

The second task includes the shared encoder and the specific classifier. The task is to preserve the local structural information, so the classifier is used to reconstruct the structure of the one-hop area of the nodes. On the other words, the classifier decodes the local structural information from the learned node embeddings based on the shared encoder so as to predict the direct neighborhoods of nodes. Thus, the adjacency matrix *A* is adopted as the classifier's expected output *Y*. The second task can be seen as a multi-label classifier task, and the loss function can be defined as follows:

(6) $$\begin{array}{r} L_{local}=-\overset{n}{\underset{i=1}{\sum }}y_{i}\text{log }{\bar{y}}_{i}+\left(1-y_{i}\right)\text{log}\left(1-{\bar{y}}_{i}\right) \end{array}$$

where ${\bar{y}}_{i}$ is the output of the classifier.

**Figure 4:**
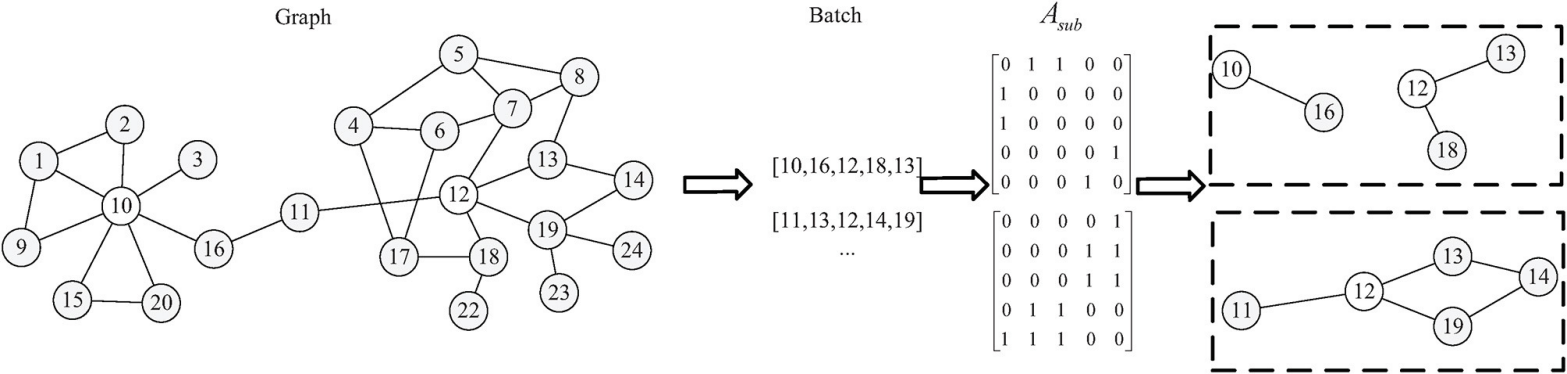
Illustration of the regularizer.

Furthermore, mini-batch batch gradient decent (MBGD) is used to optimize the parameters of the model. As shown in Figure 4, the sampled batch with a fixed number of nodes can be regarded as a sampled sub-graph. As a result of this, a regularizer component is formulated to optimize those nodes in Euclidean space so as to make nodes with edges linked closer together and nodes without edges linked farther apart.

The size of the batch is defined as *M*, and the adjacency matrix of the sampled sub-graph can be defined as $A_{sub}^{M\times M}\in A^{\left|A|\times |A\right|}$, where each element represents the relationship between nodes. We let $Z_{sub}^{|V|\times d}\in Z^{|V|\times d}$ be the corresponding sub-embedding matrix. The regularizer attempts to minimize the following contrastive loss:

(7) $$\begin{array}{l} L_{reg}=\frac{1}{2\times M\times M}\overset{M}{\underset{i=1}{\sum }}\overset{M}{\underset{j=1}{\sum }}A_{sub}\left(i,j\right)d_{i,j}^{2}\\ \begin{array}{c} \;+\left(1-A_{sub}\left(i,j\right)\right)\text{max}{\left(m-d_{i,j},0\right)}^{2} \end{array} \end{array}$$

where *m* is the margin and *d*_*i,j*_ is the Euclidean distance between the *i*-th and *j*-th representations, *d*_*i,j*_ = ||*Z*_*sub*_(*i*) − *Z*_*sub*_(*j*)||_2_.

The Euclidean distance matrix *D*, where each element represents the measure between representations, can be defined as follows:

(8) $$\begin{array}{r} \begin{array}{c} D=H+H^{T}-2G. \end{array} \end{array}$$

where *Z*_*sub*_ is Gram matrix of *G* and *H* is the Diagonal matrix of *G*.

The revised regularizer is shown as follows:

(9) $$\begin{array}{l} L_{reg}=\frac{1}{2\times M\times M}||A_{sub}⊙D\\ \begin{array}{c} \;+\left(1-A_{sub}\right)⊙\text{max}\left(m-D,0\right)|{|}_{2}. \end{array} \end{array}$$

where ⊙ is the Hadamard product.

In order to preserve the local and global structural information, we design a multi-task learning model and jointly optimize (Equations 5, 6, and 9). The objective function can be defined as follows:

(10) $$\begin{array}{r} \begin{array}{c} L=\alpha L_{global}+\beta L_{local}+\eta L_{reg}. \end{array} \end{array}$$

where α, β, and η are the corresponding weights of each task and the regularizer.

## 4. Experiments

In this section, we evaluate our proposed model, MLNE, on five real-world network datasets and three tasks, namely node classification, link prediction, and visualization. The experimental results demonstrate that MLNE has competitive performance.

### 4.1. Datasets

There are five real-world networks in our experiments, including a social network, citation networks, and a language network. They are listed as follows:

- *Cora* (McCallum et al., 2000) is a citation network with 2,708 nodes and 5,429 edges, where the nodes represent the scientific publications and the edges represent the citation relationship between publications. The nodes are split into seven classes according to scientific field.
- *DBLP* (Tang et al., 2008) is another citation network composed of 13,184 publications from five classes and with 95,955 edges.
- *20-NEWSGROUP* (Lang, 1995) is a language network that contains 20,000 newsgroup documents with 20 different labels. The tf-idf vectors of each word are adopted as the representations of documents, and cosine similarity is used to measure the similarity between documents. We select 592 documents from three classes, *com.graphics, rec.sport.baseball*, and *talk.politics.gums* respectively, to build a network in which the nodes represent the documents and cosine similarity is the weight of each edge.
- *Blogcatalog* (Tang and Liu, 2009) is a social network in which nodes represent the authors and edges represent the relationships between users. According to user interests, there are 39 different categories, and each user is labeled with at least one category. The network contains 10,312 nodes and 333,983 edges.
- *Pubmed* (Sen et al., 2008) is a citation network collected from the PubMed database in which nodes represent scientific publications and all the nodes are classified into three classes. This network consists of 19,717 nodes and 44,338 edges.

### 4.2. Baseline Algorithms

We consider the following three baseline algorithms.

- *DeepWalk* adopts random walk to sample paths composed of discrete nodes, and the Skip-gram model, which has achieved great success in word embedding, is used to generate node representations from the sampled paths.
- *node2vec* optimizes the DeepWalk through jointly combining the BFS and DFS. There are two hyperparameters, *p* and *q*, that lead the sampling such that the network structure can be deeply exploited.
- *GraRep* builds *k* different node representations by SVD and connects them so as to generate a global node representation.

### 4.3. Parameter Setting

As mentioned in Perozzi et al. (2014); Grover and Leskovec (2016), we set walk length *l* = 80, number of walks *n* = 10, and window size *w* = 10 for random walk in DeepWalk and node2vec. Specifically, we employ a grid search over return and in-out hyperparameters *p, q* ∈ {0.25, 0.5, 1, 1.5, 2} by 10-fold cross-validation for node2vec. For GraRep, we set the number of sampling steps *k* = 4 by trial and error.

In our model, the shared encoder contains an input layer and a hidden layer, where the size of the input layer is the same as the size of network *V* and the size of the hidden layer is the dimensionality of the node representation vector. The decoder and the classifier contain an output layer with the size of |*V*|. The *sigmoid* activation function is used in all layers. For hyperparameters, α, β, and η are set at 1000, 1, and 10, respectively, through using grid search on the validation set. As suggested in Cao et al. (2016), we set *K* = 10 and γ = 0.98 for random surfing.

For a fair comparison, the dimensionality of node representation vector *d* is set to 128 for all algorithms, as used in Cao et al. (2015).

### 4.4. Link Prediction

The link prediction task is to predict whether an edge exists between pairwise nodes in the original network. In order to conduct the task, a portion of the existing edges in the original network is randomly selected to be hidden. The remaining networks are then used as the input of NRL models. Node embeddings can then be learned from the trained models, and the inner product between the representation vectors of pairwise nodes is normalized by the *sigmoid* function. To evaluate the performance of each algorithm over the link prediction task, 10% of the hidden edges are utilized as the positive data. In addition, an equal number of edges not existing in the network is sampled as the negative data. AUC and Macro-F1 are utilized as evaluation metrics.

Table 1 shows the results of link prediction on Cora, 20-NEWSGROUP, and Blogcatalog. We find that MLNE and GraRep perform well but DeepWalk and node2vec have similar and poor performance. MLNE is consistently better than the baselines with respect to Macro-F1. For AUC, GraRep and MLNE perform similarly in most cases and outperform the others, except for in 20-NEWSGROUP, where GraRep is markedly better than MLNE, outperforming it by 22.71%.

**Table 1:** Macro-F1 and AUC on Cora, 20-NEWSGROUPS, and Blogcatalog for the link prediction task.

**Model**	**Cora**		**20-NEWSGROUP**		**Blogcatalog**	
	**Macro-F1**	**AUC**	**Macro-F1**	**AUC**	**Macro-F1**	**AUC**
DeepWalk	37.21	86.76	39.25	58.15	44.02	55.30
node2vec	33.33	83.22	33.95	59.88	38.20	55.81
GraRep	64.35	93.24	57.08	78.93	47.01	77.97
MLNE	80.95	93.76	60.24	64.32	68.67	77.47

### 4.5. Node Classification

Node classification is an important task in network analysis. Thus, this task is used to evaluate the quality of different learned network representations. In this experiment, *Logistic Regression* (LR) is used as a classifier. A portion of the labeled nodes are randomly selected as the training dataset, and thus the remaining nodes without labels are adopted to test the performance. The training ratio is raised from 10% to 90%. The process is repeated 10 times for all algorithms on five networks. The nodes in Blogcatalog have at least one label and thus Micro-F1 and Micro-F1 are used as with the evaluation metrics. The experimental results are reported in Tables 2–5.

**Table 2:** Node classification results on Cora.

	**Model**	**10%**	**20%**	**30%**	**40%**	**50%**	**60%**	**70%**	**80%**	**90%**
Micro-F1	DeepWalk	76.68	79.55	81.00	82.08	82.85	83.11	83.11	83.58	84.39
	node2vec	77.16	79.40	80.16	81.11	81.51	81.86	81.79	82.08	82.18
	GraRep	75.16	76.64	77.25	77.92	78.06	78.48	77.75	78.28	77.75
	MLNE	75.57	79.34	81.01	82.43	82.99	83.52	83.81	84.11	84.57
Macro-F1	DeepWalk	75.27	78.40	79.91	81.19	82.04	82.24	82.19	82.62	83.41
	node2vec	75.67	78.40	79.20	80.39	80.90	81.36	81.42	81.60	81.92
	GraRep	73.21	74.97	75.52	76.31	76.41	76.74	75.96	76.68	76.15
	MLNE	74.66	78.22	79.89	81.55	82.01	82.61	83.00	83.17	83.73

Table 2 shows the results of node classification on Cora. We find that MLNE has good performance. As the training ratio increases, MLNE outperforms the others on Macro-F1 and Micro-F1. When the training ratio is less than 50%, MLNE achieves better performance than DeepWalk and GraRep with a 90% training ratio. For Micro-F1, MLNE has the best performance in most cases. When the training ratio is better than 30%, MLNE achieves 0.39, 2.05, and 6.77% gains over DeepWalk, node2vec, and GraRep, respectively. For Macro-F1, MLNE has performance that is competitive with DeepWalk. As the training ratio increases, MLNE is better than the other baselines.

Table 3 shows the results of node classification on DBLP. The number of different labels in DBLP is lower than in the other networks, and thus the evaluation metrics of all of the algorithms are good. DeepWalk maintains a slight advantage over the others in most cases on Micro-F1 and Macro-F1. GraRep has a poor performance and it is worse than MLNE on those metrics, by 1.02 and 1.23%, respectively.

**Table 3:** Node classification results on DBLP.

	**Model**	**10%**	**20%**	**30%**	**40%**	**50%**	**60%**	**70%**	**80%**	**90%**
Micro-F1	DeepWalk	90.96	91.61	91.97	92.28	92.40	92.65	92.61	92.65	92.52
	node2vec	90.89	91.50	91.85	92.07	92.24	92.35	92.43	92.52	92.27
	GraRep	90.51	90.77	90.97	91.11	91.13	91.30	91.35	91.41	91.39
	MLNE	90.34	91.58	92.04	92.16	92.29	92.38	92.41	92.58	92.57
Macro-F1	DeepWalk	90.46	91.20	91.61	91.96	92.12	92.18	92.32	92.40	92.31
	node2vec	90.43	91.12	91.50	91.76	91.596	92.07	92.13	92.24	92.02
	GraRep	89.94	90.22	90.43	90.58	90.61	90.81	90.83	90.90	90.93
	MLNE	89.80	91.15	91.67	91.84	92.00	92.10	92.11	92.31	92.31

Table 4 shows the results of node classification on 20-NEWSGROUP. We find that MLNE has the best performance on Micro-F1 and Macro-F1. In fact, MLNE with only 10% training ratio data arrives at a result close to DeepWalk and node2vec when they are given 90% of the data. Compared with DeepWalk, the Micro-F1 values of node2vec, GraRep, and MLNE improve by 12.86, 10.29, and 3.25%. For Macro-F1, MLNE is also better than those baselines, by 13.39, 11.01, and 3.31%. DeepWalk and node2vec have similar performance and are worse than GraRep on these metrics.

**Table 4:** Node classification results on 20-NEWSGROUP.

	**Model**	**10%**	**20%**	**30%**	**40%**	**50%**	**60%**	**70%**	**80%**	**90%**
Micro-F1	DeepWalk	61.16	69.64	74.34	75.39	77.33	78.57	79.04	78.31	79.17
	node2vec	65.52	71.30	75.54	77.08	78.55	79.75	80.00	79.41	80.17
	GraRep	69.91	77.36	80.60	82.25	83.58	84.47	84.61	85.71	85.50
	MLNE	79.62	82.05	83.49	84.27	84.43	84.77	85.56	86.05	85.85
Macro-F1	DeepWalk	59.51	69.11	74.28	75.39	77.31	78.56	78.94	78.03	78.95
	node2vec	63.23	70.95	75.52	77.07	78.54	79.71	79.87	79.07	79.70
	GraRep	69.60	77.31	80.60	82.22	83.56	8445	84.49	85.56	85.33
	MLNE	79.63	82.06	83.50	84.25	84.41	84.73	85.44	85.84	85.64

Table 5 shows the results of node classification on Blogcatalog. For Micro-F1, when the training ratio is greater than 10%, MLNE is better than DeepWalk, node2vec, and GraRep, by 3.58, 3.57, and 3.68% respectively. Additionally, with a 60% training ratio of data, it beats all of the other algorithms, even when they are given a 90% training ratio. For Macro-F1, the performance of MLNE, DeepWalk, and node2vec proved much more competitive. When the training ratio is less than 60%, MLNE performs better than the baselines. As the training ratio increases from 60 to 90%, node2vec outperforms the others. GraRep has the worst performance on both metrics.

**Table 5:** Node classification results on Blogcatalog.

	**Model**	**10%**	**20%**	**30%**	**40%**	**50%**	**60%**	**70%**	**80%**	**90%**
Micro-F1	DeepWalk	33.84	36.71	38.08	38.87	39.46	39.91	40.51	40.76	41.22
	node2vec	33.83	36.49	38.09	38.95	39.67	40.04	40.34	40.96	41.02
	GraRep	36.15	38.05	38.81	39.19	39.51	39.66	39.83	39.89	40.11
	MLNE	35.74	38.85	39.83	40.58	41.15	41.34	41.64	41.80	41.87
Macro-F1	DeepWalk	19.02	22.13	23.79	24.44	25.17	25.61	26.35	26.42	26.75
	node2vec	19.71	22.77	24.63	25.69	26.43	26.80	27.13	27.75	27.70
	GraRep	19.63	22.23	22.63	23.03	23.24	23.45	23.59	23.74	24.26
	MLNE	22.27	24.40	25.39	26.16	26.44	26.56	26.78	26.85	26.94

Table 6 shows the results of node classification on Pubmed. For Micro-F1, when the training ratio is equal to 10%, Deepwalk is better than MLNE, and MLNE outperforms the other algorithms. When the training ratio is greater than 10%, the proposed algorithm MLNE outperforms all of the comparison algorithms. For Macro-F1, MLNE performs better than all of the baselines. GraRep also has the worst performance on Micro-F1 and Macro-F2.

**Table 6:** Node classification results on Pubmed.

	**Model**	**10%**	**20%**	**30%**	**40%**	**50%**	**60%**	**70%**	**80%**	**90%**
Micro-F1	DeepWalk	80.06	80.80	80.89	80.97	81.01	80.55	80.49	80.10	80.83
	node2vec	79.23	80.03	80.16	80.42	80.21	79.91	79.78	79.87	80.83
	GraRep	79.14	79.39	79.39	79.78	79.66	79.56	79.51	78.83	79.82
	MLNE	80.03	81.09	81.44	81.55	81.62	81.71	81.76	81.82	82.35
Macro-F1	DeepWalk	78.69	79.44	79.54	79.69	79.69	79.19	79.06	78.81	79.78
	node2vec	77.70	78.53	78.61	78.96	78.63	78.40	78.16	78.52	79.78
	GraRep	77.70	78.00	77.98	78.53	78.35	78.18	78.18	77.56	78.83
	MLNE	78.73	79.78	80.12	80.24	80.32	80.42	80.44	80.52	81.11

### 4.6. Visualization

Visualization is another important task for exploring and analyzing a network. To conduct this task, the size of learned node embeddings is firstly reduced for display; a popular dimensionality reduction technique *t*-SNE is used to visualize the network in two-dimensional space. For documents labeled into three categories in the 20-NEWSGROUP, three different colors indicate the corresponding points. A good visualization result keeps nodes within the same cluster close and vice versa.

**Figure 5:**
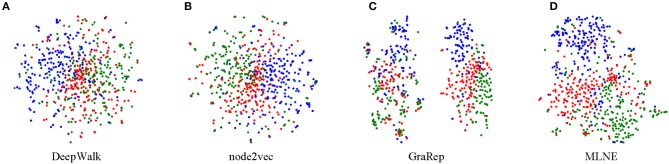
Visualization of 20-NEWSGROUP. Each point represents a document. The color indicates the category of each document. **(A)** is the result of DeepWalk, **(B)** is the result of node2vec, **(C)** is the result of GraRep, and **(D)** is the result of the proposed MLNE.

From Figure 5, we can see that DeepWalk and node2vec do not perform well because there are no clear boundaries among the groups. For GraRep, there are two clusters where nodes also tend to mix together. Obviously, MLNE slightly outperforms the baselines and learns a good clustering, resulting in much clearer boundaries. The experimental results demonstrate the effectiveness of MLNE in the visualization task.

## 5. Conclusion

In this paper, we propose a multi-task learning-based network embedding named MLNE. In order to jointly preserve the local and global structural information, we design a model based on multi-task learning. The model is composed of three components: a shared encoder, decoder, and classifier. The shared encoder and decoder can be seen as a standard autoencoder that automatically learns representations from the global features. The shared encoder and classifier are used to reconstruct the one-hop area of a node from the learned latent representation. Additionally, a regularization based on mini-batch batch gradient descent is introduced to learn stable and robust representations. Experimental results on node classification, link prediction, and visualization tasks demonstrate the superiority of our proposed MLNE in learning node representations.

In the future, we will extend multi-task learning to heterogeneous information networks and large-scale networks.

## Data Availability Statement

The dataset *Cora* for this study can be found at https://linqs.soe.ucsc.edu/node/236. The dataset *DBLP* for this study can be found at http://arnetminer.org/citation. The dataset *20-NEWSGROUP* for this study can be found at http://qwone.com/~jason/20Newsgroups/. The dataset *Blogcatalog* for this study can be found at http://socialcomputing.asu.edu/datasets/BlogCatalog3. The dataset *Pubmed* for this study can be found at https://linqs.soe.ucsc.edu/data.

## Author Contributions

SW and MG designed the experiments. QW performed the experiments. SW and QW analyzed the data. SW, MG, and QW wrote the paper.

### Conflict of Interest

The authors declare that the research was conducted in the absence of any commercial or financial relationships that could be construed as a potential conflict of interest.

## References

[B1] Argerich L. Zaffaroni J. T. Cano M. J. (2016). Hash2vec, feature hashing for word embeddings. arXiv [preprint] arXiv:1608.08940.

[B2] Belkin M. Niyogi P. (2002). Laplacian eigenmaps and spectral techniques for embedding and clustering, in Advances in Neural Information Processing Systems (Vancouver, BC), 585–591.

[B3] Cao S. Lu W. Xu Q. (2015). Grarep: learning graph representations with global structural information, in Proceedings of the 24th ACM International on Conference on Information and Knowledge Management (New York, NY: ACM), 891–900.

[B4] Cao S. Lu W. Xu Q. (2016). Deep neural networks for learning graph representationsm, in Thirtieth AAAI Conference on Artificial Intelligence (Phoenix, AZ), 1145–1152.

[B5] Chen H. Perozzi B. Al-Rfou R. Skiena S. (2018). A tutorial on network embeddings. arXiv [preprint] arXiv:1808.02590.

[B6] Donnat C. Zitnik M. Hallac D. Leskovec J. (2018). Learning structural node embeddings via diffusion wavelets, in Proceedings of the 24th ACM SIGKDD International Conference on Knowledge Discovery & Data Mining (New York, NY: ACM), 1320–1329.

[B7] Du B. Wang S. Xu C. Wang N. Zhang L. Tao D. (2018). Multi-task learning for blind source separation. IEEE Trans. Image Process. 27, 4219–4231. 10.1109/TIP.2018.2836324 29870343

[B8] Fortunato S. (2010). Community detection in graphs. Phys. Rep. 486, 75–174. 10.1016/j.physrep.2009.11.002

[B9] Gong M. Cai Q. Ma L. Wang S. Lei Y. (2017). Computational Intelligence for Network Structure Analytics. Singapore: Springer.

[B10] Goyal P. Ferrara E. (2018). Graph embedding techniques, applications, and performance: a survey. Knowledge Based Syst. 151, 78–94. 10.1016/j.knosys.2018.03.022

[B11] Grover A. Leskovec J. (2016). node2vec: scalable feature learning for networks, in Proceedings of the 22nd ACM SIGKDD International Conference on Knowledge Discovery and Data Mining (New York, NY: ACM), 855–864. 10.1145/2939672.2939754 PMC5108654 27853626

[B12] Hamilton W. L. Ying R. Leskovec J. (2017). Representation learning on graphs: methods and applications. arXiv [preprint] arXiv:1709.05584.

[B13] Herman I. Melançon G. Marshall M. S. (2000). Graph visualization and navigation in information visualization: a survey. IEEE Trans. Visual. Comput. Graph. 6, 24–43. 10.1109/2945.841119

[B14] Hu R. Aggarwal C. C. Shuai M. Huai J. (2016). An embedding approach to anomaly detection, in IEEE International Conference on Data Engineering (Helsinki), 385–396.

[B15] Lang K. (1995). Newsweeder: learning to filter netnews, in Machine Learning Proceedings 1995 (Tahoe, CA: Elsevier), 331–339.

[B16] Liu X. He P. Chen W. Gao J. (2019a). Multi-task deep neural networks for natural language understanding. arXiv [preprint] arXiv:1901.11504.

[B17] Liu X. Murata T. Kim K.-S. Kotarasu C. Zhuang C. (2019b). A general view for network embedding as matrix factorization, in Proceedings of the Twelfth ACM International Conference on Web Search and Data Mining (New York, NY: ACM), 375–383.

[B18] Lü L. Medo M. Yeung C. H. Zhang Y.-C. Zhang Z.-K. Zhou T. (2012). Recommender systems. Phys. Rep. 519, 1–49. 10.1016/j.physrep.2012.02.006

[B19] Lü L. Zhou T. (2011). Link prediction in complex networks: a survey. Phys. Stat. Mech. Appl. 390, 1150–1170. 10.1016/j.physa.2010.11.027

[B20] McCallum A. K. Nigam K. Rennie J. Seymore K. (2000). Automating the construction of internet portals with machine learning. Inform. Retriev. 3, 127–163. 10.1023/A:1009953814988

[B21] Mikolov T. Chen K. Corrado G. Dean J. (2013). Efficient estimation of word representations in vector space. arXiv [preprint] arXiv:1301.3781.

[B22] Musiał K. Juszczyszyn K. (2009). Properties of bridge nodes in social networks, in International Conference on Computational Collective Intelligence (Wroclaw: Springer), 357–364.

[B23] Peng C. Xiao W. Jian P. Zhu W. (2019). A survey on network embedding. IEEE Trans. Knowledge Data Eng. 31, 833–852. 10.1109/TKDE.2018.2849727

[B24] Perozzi B. Al-Rfou R. Skiena S. (2014). Deepwalk: online learning of social representations, in Proceedings of the 20th ACM SIGKDD International Conference on Knowledge Discovery and Data Mining (New York, NY: ACM), 701–710.

[B25] Qiu J. Dong Y. Ma H. Li J. Wang C. Wang K. (2019). Netsmf: large-scale network embedding as sparse matrix factorization, in The World Wide Web Conference (New York, NY: ACM), 1509–1520.

[B26] Qiu J. Dong Y. Ma H. Li J. Wang K. Tang J. (2018). Network embedding as matrix factorization: unifying deepwalk, line, pte, and node2vec, in Proceedings of the Eleventh ACM International Conference on Web Search and Data Mining (New York, NY: ACM), 459–467.

[B27] Ruder S. (2017). An overview of multi-task learning in deep neural networks. arXiv [preprint] arXiv:1706.05098.

[B28] Sen P. Namata G. Bilgic M. Getoor L. Galligher B. Eliassi-Rad T. (2008). Collective classification in network data. AI Magaz. 29:93 10.1609/aimag.v29i3.2157

[B29] Shi C. Hu B. Zhao W. X. Philip S. Y. (2018). Heterogeneous information network embedding for recommendation. IEEE Trans. Knowledge Data Eng. 31, 357–370. 10.1109/TKDE.2018.2833443

[B30] Tang J. Qu M. Wang M. Zhang M. Yan J. Mei Q. (2015). Line: large-scale information network embedding, in Proceedings of the 24th International Conference on World Wide Web (Geneva: International World Wide Web Conferences Steering Committee), 1067–1077.

[B31] Tang J. Zhang J. Yao L. Li J. Zhang L. Su Z. (2008). Arnetminer: extraction and mining of academic social networks, in Proceedings of the 14th ACM SIGKDD International Conference on Knowledge Discovery and Data Mining (New York, NY: ACM),990–998.

[B32] Tang L. Liu H. (2009). Relational learning via latent social dimensions, in Proceedings of the 15th ACM SIGKDD International Conference on Knowledge Discovery and Data Mining (New York, NY: ACM), 817–826.

[B33] Thung K.-H. Wee C.-Y. (2018). A brief review on multi-task learning. Multi. Tools Appl. 77, 29705–29725. 10.1007/s11042-018-6463-x

[B34] Tsoumakas G. Katakis I. (2007). Multi-label classification: an overview. Int. J. Data Warehous. Mining 3, 1–13. 10.4018/jdwm.2007070101

[B35] Wang D. Cui P. Zhu W. (2016). Structural deep network embedding, in Proceedings of the 22nd ACM SIGKDD International Conference on Knowledge Discovery and Data Mining (New York, NY: ACM), 1225–1234.

[B36] Wang N. Wang H. Jia Y. Yin Y. (2018). Explainable recommendation via multi-task learning in opinionated text data, in The 41st International ACM SIGIR Conference on Research & Development in Information Retrieval (New York, NY: ACM), 165–174.

[B37] Wang X. Cui P. Wang J. Pei J. Zhu W. Yang S. (2017). Community preserving network embedding, in Thirty-First AAAI Conference on Artificial Intelligence (San Francisco, CA), 203–209.

[B38] Wei X. Xu L. Cao B. Yu P. S. (2017). Cross view link prediction by learning noise-resilient representation consensus, in Proceedings of the 26th International Conference on World Wide Web (Geneva: International World Wide Web Conferences Steering Committee), 1611–1619.

[B39] Zhang D. Han J. Yang L. Xu D. (2018). Spftn: a joint learning framework for localizing and segmenting objects in weakly labeled videos. IEEE Trans. Pattern. Anal. Mach. Intell. [Epub ahead of print]. 10.1109/TPAMI.2018.2881114 30442600

[B40] Zhang D. Yin J. Zhu X. Zhang C (in press). Network representation learning: a survey. IEEE Trans. Big Data. 10.1109/TBDATA.2018.2850013

